# Mixed methods evaluation of an employer-led, free lunch initiative in Northern Ireland

**DOI:** 10.1186/s40795-019-0321-8

**Published:** 2019-12-18

**Authors:** Désirée Schliemann, Michelle C. McKinley, Jayne V. Woodside

**Affiliations:** Centre for Public Health, Queen’s University Belfast, Institute of Clinical Sciences, Block B, Royal Victoria Hospital, Grosvenor Road, Belfast, BT12 6BA UK

**Keywords:** Workplace, Diet intervention, Free lunch, Employees, Diet, Health, Interviews

## Abstract

**Background:**

The objective of this study was to evaluate the acceptance of an employer-led free lunch initiative and its effect on health, diet, and attitudes towards health and diet amongst employees in a small workplace in Northern Ireland.

**Methods:**

This was a controlled, employer-led pilot intervention, which was evaluated through a mixed methods approach.

**Results:**

Seventeen participants from the intervention site and 14 participants from the control site completed all assessments. Post-intervention, there was no difference in change in dietary measures between the sites, except for saturated fat intake during weekdays (IS: − 1.3% of calories, SD: 4.3; CS: 2.8% of calories, SD: 6.6; *P*-value < 0.05). Qualitative information was summarised to highlight employees’ expectations and experiences with the intervention.

**Conclusion:**

This study highlights the challenges that need to be considered when implementing a free lunch initiative for staff.

## Background

Numerous health issues are associated with consuming a diet high in non-milk extrinsic sugars (NMES), saturated fatty acids (SFA), excessive calories (kcal) and salt and low in fruit (F) and vegetables (V), such as cardiovascular disease, diabetes and cancer [1]. Research suggests that employers may play an important role in facilitating healthy eating and that management support seems key for workplace wellbeing interventions to be successful [2, 3]. There are currently no guidelines in Northern Ireland (NI) that regulate workplace food policies, other than that communal eating facilities must be provided where employees may take their break, including facilities for accessing hot beverages (kettle or vending machines). In addition, guidelines from the Health and Safety Executive recommend ‘w*here hot food cannot be obtained in or reasonably near to the workplace, workers may need to be provided with a means for heating their own food (e.g. microwave oven)*’ [4] and current practices are not well studied. One survey examining lunch practices in the United Kingdom (UK) by Altman & Baruch looked at the cultural importance of lunches and described them ‘as a mirror of a company’s values’ [5]. The survey reported that 53% out of 170 organisations in the UK (≥175 staff) had facilities where full lunches were provided. Other companies had the option to purchase snacks, while some companies did not provide any on-site food, and lunches were the responsibility of the employees alone. Whether canteen lunches are generally of higher nutritional value (i.e. less SFA, NMES, salt, kcal and more F, V and fibre) than packed lunches has not yet been explored for workplace settings in the UK. However, research suggests that Finish staff who eat canteen-lunches make food choices more closely resembling dietary recommendations compared to staff eating packed lunches [6]. Evidence from systematic reviews looking at dietary behaviour change in middle-aged adults suggests that barriers to healthy eating include: a lack of time to prepare food from scratch, lack of accessibility to healthier options, misinterpretation of health messages, social eating context, lack of planning and convenience [7]. Qualitative and quantitative studies also reported that cost, choice and availability mainly influenced employees’ food choice in the workplace and that preparing food at home helped staff to eat more healthily at work [8, 9]. The aim of this study was to evaluate the acceptance of an employer-led initiative to provide free, healthy work lunches and their effectiveness in terms of improving health, diet, and attitudes to health and diet amongst employees in a small workplace in NI. The primary endpoint of this study was change in F and V intake (gram/day), both, at weekends (OFF duty) and during weekdays (ON duty) between the intervention site (IS) and control site (CS). Secondary outcomes that were assessed were change in (1) overall diet and eating habits, (2) health measures, and (3) job satisfaction between the IS and CS as well as (4) attitudes towards diet and health and (5) acceptability of the lunches and the overall initiative post-intervention by employees from the IS.

## Methods

**Fig. 1:**
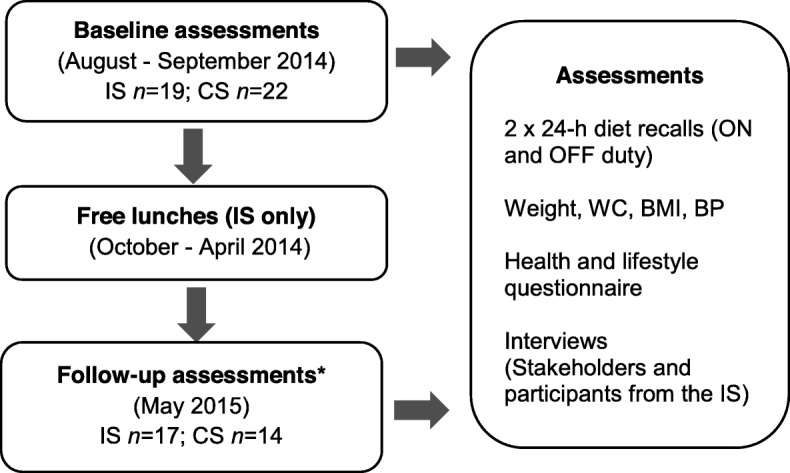
Study design and evaluation outcomes of the free lunch pilot intervention. IS- Intervention Site; CS – Control Site; ON duty – weekdays; OFF duty – weekends, WC – Waist Circumference; BMI – Body Mass Index; BP – Blood pressure. *At follow-up, two participants left the IS without giving reason. From the CS, five participants had left the company; two were following a strict diet and one dropped out without giving reason

This was a controlled, workplace-led pilot intervention to evaluate the effect of free, healthy lunches on employees’ F and V intake, overall diet and health status (Fig. 1).

### Setting

The workplace was a large carpet manufacturer operating on two worksites (one small site with 20 employees and one large site with 300 employees) both located in a rural area of NI and with sister companies worldwide. Both Northern Irish worksites did not have a canteen to start with and employees were consuming packed lunches or lunches purchased outside of work.

### Study population

In August 2014, all employees from the small site, i.e. the IS, were invited to participate and an equal number of matched controls (matched on age, gender and work type) were recruited from the large worksite, i.e.the CS (Fig. 1). Participants had to work full-time on either site as only full-time employees stay for lunch at the canteen. Furthermore, participants were excluded if pregnant, breastfeeding or following a strict diet during the intervention period as eating habits are likely not reflective of the general working population. As an incentive to participate, employees were offered personalised nutrition advice (based on the *Eatwell plate* [10], as the basis of current dietary recommendations at the time) by a researcher who was a qualified nutritionist, after completion of the study. Written consent was obtained from all participants prior to study commencement.

### Employer-led intervention

Based on the feedback from a sister company in Denmark, where a healthy meal and salads for lunch were provided for employees on a daily basis, the employer decided to pilot the provision of healthy lunches for employees at the IS, with the aim of eventually rolling it out to the control site CS. Starting late October 2014, lunches were designed and prepared by a member of catering staff who was not trained in nutrition and selected the meals on offer based on the assessment of their personal opinion of a nutritious lunch. The food was presented in a self-catered buffet type format with a choice of a hot meal, soup or sandwich, salads and fruits for a six-month trial period. The lunches were provided free of charge.

### Measurements

Employees who agreed to take part were invited for two 20 to 30 min appointments at baseline and at 6 months for a follow-up. These were scheduled by the site manager and conducted during working hours by the researcher using similar methods as described previously [11], but are summarised below.

#### Dietary measures

Two 24-h (24-h) diet recalls were conducted pre- and post-intervention to quantitatively assess nutrient intake over a 24-h period. To account for differences between OFF duty and ON duty eating habits, recalls were taken Mondays and on one other weekday. The 24-h diet recall was an adapted version of the validated UK standard 24-h diet recall as used previously for workplace diet interventions [11, 12]. All diet recalls were entered using the dietary analysis software package WISP (Weighed Intake Software Program; Tinuviel Software, Warrington, UK). Dietary information was transferred into SPSS to analyse the main nutrients of interest (i.e. kcal, energy from total fat (Fat), SFA, NMES, F and V intake). To reduce inter-observer error, baseline and follow-up recalls were entered by one researcher and checked for accuracy by a second researcher.

#### Health measures

Body mass index (BMI) was calculated based on weight and height measurements taken during the study visits (kg/m^2^). To calculate waist circumference (WC), the average distance between the last rib and the hipbone was taken and from there the midway WC was measured. Systolic (SBP) and diastolic blood pressure (DBP) readings were assessed using an OMRON M5–1 digital blood pressure monitor. All readings were conducted three times to increase accuracy and the average was calculated from the second and third reading.

#### Study questionnaires

A modified version of a validated questionnaire [12] was used to assess demographic characteristics, working habits and general health as well as diet behaviour and knowledge [13], physical activity [14], smoking and alcohol consumption [15] and job satisfaction [16].

#### Interviews

Semi-structured interviews were carried out with stakeholders and employees to assess their motivation and views on healthy eating at work and the acceptability of the lunches. The topic guide was developed by the research team with questions that were adapted from previous qualitative studies [8, 11, 17] (Additional file 1). Questions regarding improvement suggestions were included at follow-up. The topic guide was piloted and probes were added that were specific to the workplace, e.g. referring to vending machines and break time as potential barriers to eat healthy. Similarly, follow-up questions were tailored post-intervention to capture workplace specific issues (e.g. tidiness and portion sizes). Interviews with stakeholders who were involved with organising the food court were similar to employee interviews and also included questions assessing their motivation to provide healthy lunches. The interviews were conducted by one researcher (DS) who met interviewees only briefly prior to the interviews (i.e. for conducting the dietary recall and health measurements). All interviews took 20 to 30 min and were tape-recorded and transcribed anonymously into Microsoft Word. The transcripts were analysed using thematic analysis [18] by one researcher (DS) who was trained in qualitative research and confirmed by a second researcher (JVW). Codes and candidate themes were identified by looking across the data and grouped into main themes.

#### Statistical analysis

Quantitative data was analysed with the statistical software package SPSS version 20. The change in diet- and health-related endpoints was compared between IS and CS using student independent samples t-tests for continuous variables and Chi-square test for categorical variables. Change within sites was analysed using paired samples t-test for continuous variables and Chi-square test for categorical variables.

## Results

Nineteen participants from the IS (10 male and 9 female) and 22 participants from the CS (11 male and 11 female) completed all baseline assessments. There was a higher proportion of manual workers in the IS (73.3%) compared to the CS (59.1%). At follow-up, two people from the IS and five participants from the CS dropped out (Fig. 1). The analysis, therefore, included 17 participants from the IS and 14 from the CS.

### Diet

At baseline, the CS consumed significantly more F than the IS ON duty (IS: 187.9 g, SD: 170.2; CS: 332.1 g, SD: 237.8; *p*-value ≤0.05) (Table 1). Participants from both sites consumed more F than V. Participants from the IS and CS on average met the kcal recommendations ON and OFF duty, however, OFF duty, both sites consumed more kcal and SFA compared to ON duty. The IS consumed more SFAs than recommended, both ON and OFF duty and significantly more than the CS ON duty (IS: 13.9% of kcal, SD: 3.8; CS: 10.1% of kcal, SD: 3.5; *p*-value < 0.01). Participants from both sites, on average, reported a notably higher intake of NMES than recommended, both, ON and OFF duty. The CS met the daily recommended values (DRVs) guidelines, ON and OFF duty, for total fat consumption, whereas the IS consumed slightly more than recommended.

**Table 1:** Difference in dietary change at follow-up (ON and OFF duty) between the IS (*n* 17*)* and CS (*n* 14)

		IS OFF duty		IS ON duty		CS OFF duty		CS ON duty		*P*-value	*P*-value
		Baseline	Change^a^	Baseline	Change^a^	Baseline	Change^a^	Baseline	Change^a^	OFF ^b^	ON^c^
Calories^d^ (kcal)	Mean SD	1886.8 794.5	−152.0 718.0	1767.8 621.4	−114.7 581.4	2117.0 1118.2	−0.7 792.6	2005.0 674.7	−94.5 561.8	0.58	0.92
Total Fat (% of kcal)	Mean SD	35.1 6.7	0.5 9.2	35.1 7.1	−0.6 10.4	31.8 10.2	1.8 12.5	32.9 9.1	−0.5 13.0	0.74	0.97
SFA (% of kcal)	Mean SD	15.4 4.1	−2.3 5.7	13.9 4.0	−1.3 4.3	13.5 6.0	0.5 5.0	10.3 3.4	2.8 6.6	0.23	< 0.05
NMES (% of kcal)	Mean SD	10.9 8.4	−1.1 4.5	6.7 6.1	4.1 7.3	12.4 9.2	3.4 8.7	7.7 5.9	6.6 11.3	0.08	0.47
Sodium (mg)	Mean SD	2617.5 1243.0	− 307.7 411.4	3045.3 1477.7	− 440.5 1821.4	3029.2 1416.8	165.3 2088.5	3224.2 1624.0	− 121.2 2378.9	0.49	0.68
F (g)	Mean SD	156.6 171.8	− 60.7 133.8	148.1 202.9	77.4 163.8	250.0 220.3	− 102.1 304.2	284.2 238.3	−6.4 324.3	0.64	0.36
V (g)	Mean SD	124.8 139.6	48.9 204.8	114.0 86.8	−4.8 121.3	196.4 134.8	−19.6 176.1	187.4 183.3	−2.1 257.2	0.33	0.97

OFF duty – weekends, ON duty – weekdays, *CS* control site, *IS* intervention site, *SFA* saturated fatty acid, *NMES* non-milk extrinsic sugars, *kcal* calories, *mg* milligram, *g* gram, *F* Fruit, *V* Veg

The findings in this table are presented for participants who completed the follow-up assessments only

^a^ Values demonstrate the change at follow-up within the IS and CS, separately

^b^ *P*-values demonstrate the statistically significant difference in change at follow-up in OFF duty eating habits between the IS and CS

^c^ *P*-values demonstrate the statistically significant difference in change at follow-up in ON duty eating habits between the IS and CS

^d^ Calories refers to the total number of calories consumed in one day

At follow-up (Table 1), the IS significantly reduced their ON duty SFA intake compared to the CS (IS: − 1.3% of kcal, SD: 4.3; CS: 2.8% of kcal, SD: 6.6; *P*-value < 0.05). Change in F intake ON duty was slightly higher in the IS at follow-up (77.4 g, SD: 163.8 g) and lower in the CS (− 6.4 g, SD: 324.3 g). V intake stayed the same ON duty, however, any change in F or V intake was not significant. There was no significant difference in change in any other dietary measures between the two sites ON and OFF duty.

### Health

Table 2 displays baseline and change data and highlights that there was no significant difference between IS and CS at baseline and there was no difference in change in health measures between the sites (data not shown for smoking, alcohol consumption and physical activity). On average, participants from both sites were classified as overweight at baseline and at follow-up.

**Table 2:** Difference in change in health measures within and between the IS and CS

		IS (*n* 17)		*P*-value^a^	CS (*n* 14)		*P*-value^a^	IS vs CS *P*-value^b^
		Baseline	Change		Baseline	Change		
*Change in health measures and EQ-5D score*								
Age	Mean SD	44.6 8.4	–	–	47.7 7.6	–	–	0.30
Job type office	N (%)	4 (23.5)	–	–	4 (28.6)	–	–	0.75
manual	N (%)	13 (76.5)	–	–	10 (71.4)	–		
Height (cm)	Mean SD	165.0 8.4	–	–	168.6 8.5	–	–	0.24
Weight (kg)	Mean SD	80.7 23.9	−0.4 1.5	0.32	75.4 14.2	0.6 2.0	0.24	0.11
BMI (kg/m^2^)	Mean SD	29.4 7.7	−0.09 1.0	0.71	26.4 3.8	0.3 0.7	0.22	0.28
WC (cm)	Mean SD	93.4 20.6	0.9 4.6	0.43	89.8 13.0	0.3 3.3	0.70	0.71
SBP mmHg	Mean SD	124.1 11.4	−0.3 17.8	0.95	130.6 14.1	−4.9 10.3	0.10	0.40
DBP mmHg	Mean SD	75.7 8.6	−0.1 13.7	0.97	79.3 12.4	−2.0 6.4	0.25	0.62
EQ-5D (total)	Mean SD	23.9 1.5	0.2 0.7	0.27	24.4 1.4	0.2 0.7	0.27	0.92
EQ-5D (%)	Mean SD	75.9 13.1	−4.1 13.8	0.24	83.2 9.5	1.0 12.3	0.78	0.31

*CS* control site, *IS* intervention site, *cm* centimetres, *BMI* body mass index, *WC* waist circumference, *SBP* systolic blood pressure, *DBP* diastolic blood pressure

The findings in this table are presented for participants who completed the follow-up assessments only

^a^ *P*-values demonstrate the statistically significant difference in change at follow-up within the IS and CS

^b^ *P*-values demonstrate the statistically significant difference in change at follow-up between the IS and CS (except for job type, age, height where *P*-value demonstrates difference between sites pre-intervention)

### Job satisfaction

There was no significant change in job satisfaction score pre- and post-intervention and no difference between the two sites post-intervention despite the introduction of the free lunches at the IS (data not shown).

### Interviews

#### Baseline

Employees (*n* 10) and four key stakeholder were interviewed at baseline. Key themes discussed by staff during the baseline interviews when asked about their current diet, influences on food choice at home and at work and about their opinions on the food court are demonstrated in Table 3. Employees had a basic understanding about healthy eating and were hoping to learn more as a result of the food court. Before the food court was introduced, employees were either bringing in packed lunches in form of sandwiches, dinner leftovers or take-away meals. Some reservations were voiced about the proposed food court and are highlighted in Table 3. Suggestions and concerns expressed by employees were shared anonymously with management to be addressed from the start.

**Table 3:** Employee interviews from the intervention site at baseline

Pre-intervention interviews		
Key-themes	Sub-themes	
Healthy eating & Health	Influenced by family members	*‘Probably since I’ve had my son, my priority has changed to seeing that he’s ok then if I’m ok and then I had an ill partner as well so he required a lot of attention. So a lot of the time it was just grabbing things on the go you know it was too much time to sit down and cook a meal […].’* Female
	Basic understanding	*‘Yeh, ehm, well I think the salt can actually clock up your arteries and well fat it makes your heart work harder and you know things like that.’* Female
	Desire to get a better understanding	*‘Oh, I’d love to learn you know a wee bit more stuff like that there you know […] eat the right stuff you know years ago they used to say ‘a brandy at night and you’ll be alright. You know or you go to these Chinese shops these tablets here vitamins take one a day, olive oil or whatever, stuff like there that but then nobody has ever come back and said you know they are good for you are they’re not good for you.’* Male
	Influenced by social gatherings	*‘[…] I would go to a lot of football matches and we go to football games and you know it’s all […] chip vans and they’re not healthy you know they’re in front of you and you feel hungry what do you do?’* Male
Current lunch practice	Packed lunches (sandwiches or takeaway meals)	*‘[…] my wife she prepares it. What way do I put it? I try not to – whenever she’s making sandwiches you know it’s not white bread, its wholemeal bread. […]Because she knows I am conscious of what I should eat.’* Male
	Food choice influenced by convenience, (limited) availability, (short) break time, flavour	*‘They only give you, you come in at eight o’clock and you get half hour. You get one half hour for that whole day […] and the half hour you spent trying to get as much in to you, to do you until you get home that night.’* Female
	Cost is no issue	*‘[…] if you go to the fridge and readymade meals you know for example beef curry and rice and then there’s sitting beside beef curry a rice low fat, I pick the low fat one you know I would do that and this costs more it’s actually maybe twice the price believe it or not.’* Male
Lunch Environment	Cold lunch room, especially in winter	*‘The canteen like I say it would be nice for, to have a bit of comfort to enjoy, try and enjoy your dinner but if you don’t have heating and you have hard plastic cold seats to sit on ehm, it’s not nice especially if you only get one break in a day.’* Male
	Not very inviting	
	Limited facilities	*‘There’s ten people going in – half an hour and there’s two microwaves. Ten people to get through in half an hour and a microwave maybe two minutes time you can’t even do that.’* Male *‘[…] there should be more meals in work […] I sometimes I bring my own meals and sometimes but often just revert to sandwiches you know but its not very healthy like even sandwiches every day cause then you’re - you’re working and you’re hungry again after that and tend to you know maybe go for a sweet bar or something like that from the machine stuff cause you feel a wee bit sugarish coming on […].’* Male
	Lack of healthy options due to location	
	Vending machines are a temptation	
Company’s Responsibility	Lunches are not the responsibility of the employer (some agree/ some disagree)	*‘Yes of course, because I mean it gets promotion in the schools and so forth so why not in the workplace to keep it going.’* Female *‘I don’t think they’re responsible for it you know cause everybody sort of can eat what they want but you know it certainly doesn’t do anyone any harm trying to maybe change people’s mind-sets on what’s healthy and what’s not.’* Male
Concerns about Food Court	Not enough supply (last people go hungry) Healthiness of the food	*‘I think there are worries that the last one in the queue won’t get anything and maybe they have possibly nothing else with them so you don’t want anyone not to have any lunch so I think it just needs to be put in place to ensure that there is enough food for everyone.’* Female *‘[…] if they’re thinking of bringing food in you don’t know whether it’s been processed and stuff like that you know you would like to see it you know a canteen is fresh, they do fresh stuff there they cook it there and then they don’t have you know processed food and stuff like that. Processed foods contain a lot of additives you know and it’s not healthy you know.’* Male
Suggestions for food court	Light lunch	*‘It’s a lighter lunch that we should be having because when people are working hard all day and they go home in the evenings their dinner is their main meal […].’* Female
	Seasonality important (hot food in winter, lighter foods in summer)	*‘I think it has to be seasonal you know I have salads throughout the summer but coming to the cooler weather I want something warm.’* Female
	Variety of different foods	*‘[…] the food would need to be a different variety. It can’t be the same food all the time.’* Female
	Healthy snacks	*‘Maybe if there was fruit, like a fruit machine. […] it’s just not readily available and it’s, I don’t want to go to the shop and get a banana.’* Male
	Hygiene (messy and unappealing)	*‘[…] if it was like a buffet thing – they would eat all round them and pick through it and get on and that would turn an awful lot of people of […].’* Female
	Not meeting everyone’s taste	*‘Well if there was food provided then again you know its hard catering for a lot of different tastes.’* Male
Learning from intervention	Learn about healthy eating	*‘[…] everybody sort of can eat what they want but you know it certainly doesn’t do anyone any harm trying to maybe change people’s mindsets on what’s healthy and what’s not. To be honest we – you know people don’t really know what they’re eating. You don’t know what’s good for you and what’s bad for you – you know we haven’t been sort of educated so that’s why I was keen for this too – maybe I learn something it’ll be a benefit to me.’* Male
	Improve eating habits as result	

When the stakeholders were asked about the motivations of the company to implement the food court, mixed messages were given about its purpose and expectations on the food court in terms of nutrition:

*‘[…] when we as a company – we are knowledgeable that healthy eating is important and the benefits to health, the benefits to our employees’ health is very important to us because we need people here. You know and we need them to be healthy and enjoying their work and to provide healthy choices’ Stakeholder 1*

*‘[…] we’re not on a sort of campaign of making everybody – of making everybody into healthy eaters you know we’re here primarily to run a business. But if healthy eating means people have less absence that obviously has a bottom line impact for us. […] The idea is we are introducing a food court providing food service for people. Not all healthy you know it’s not about just, ehm, healthy […].’ Stakeholder 2*

#### Follow - up

Eleven employees (including employees who completed the baseline interviews and others who did not) from the IS and three of the same stakeholders were interviewed at follow-up. All of the staff reported that they had tried the meals from the food court for at least one to two months when it was introduced. Since then, a number of people had stopped having their lunch from the food court completely (*n* 6), a few were having the lunch provided, but not every day (*n* 3), and around half of the staff were making use of the food court on a daily basis (*n* 9). Table 4 presents clear themes and sub-themes that emerged of positive and negative experiences that staff had with the food court.

**Table 4:** Employee interviews from the intervention site at follow-up

Post-intervention interviews		
Key-themes	Sub-themes	Quotes
Food handling and sourcing	Food handling practices of some staff members	*‘The hygiene, I would be a very picky eater, and that’s just me personally, but on two occasions I’ve seen two people licking the knife and putting it into the butter. Now that does me, that’s me finished. […] Another thing, whenever you’re on the late shift the food is sitting out from 12 o’clock to 2 o’clock and flies, and we’re now coming into the time when there’s flies, I seen flies. The hygiene, just people doing things that I wouldn’t want done, that I would be easy put off, so, therefore, that would just finish me.’* Female
		*‘If it was just down to her, personally, I think it would be far better, it would be more of a success, I think. There’s too many people handling food and trying to ... not putting food away properly and not storing it properly and just that.’ Female*
	Deteriorating quality of food	*‘They’re going for [value supermarket] now and the theory I think behind it is, they’re getting a freezer and they’re buying the deals, 2 for 1. Frozen pies from [the value supermarket] you don’t know what’s in it.’* Female
	Food waste	*‘Whenever the food court is going on, there’s an awful lot of food actually thrown out, so there is. When there is soup, and they don’t like soup, they’re not having it.’* Female
Food	Good availability of food Enjoy the food served	*‘It’s been dead on, it’s been great. Sometimes I would still bring a sandwich, even I’m getting the food court, if I start that bit earlier, and sometimes I would say that I’m eating too much […] But, as I say, I’m happy enough with it, I think it’s dead on.’* Male
Staff	Service staff friendly and accommodating	*‘There was nice looking pie there but I wouldn’t really call it healthy, but if you take a reasonable slice and not too much of the bread with it, pie and bread, you know, pie and maybe a side salad, you know. But the food did taste, [the lady serving the food] is actually doing a good job.’*Female
Environment	Improved Facilities (new look and heating)	*‘It’s always a bit cleaner. We had a wee bit of bother with the heat in winter there. Sometimes you were going in and maybe one heater, but they’ve got the heating on in it now, you feel more comfortable.’* Male
Social aspect	Encouraged office workers to have lunch in the communal eating facilities	*‘We would have probably always ate at our desks before, which now we’re using the canteen, which I think is definitely better. It’s a bit more social and get away from the computer and everything, so I do think that’s a big positive that has come out of it, definitely.’* Female
Lack of education	Feeling of being misled at the start, expected to learn about healthy eating	*‘There have been an awful lot of wires crossed. It’s not what I think it would be. We were really looking forward to it, to what we would be educated on […] to what we would be eating.’* Female
	Expectations on nutritional quality and portion control were not met - disappointment	*‘I don’t think they were changes for the good, so I don’t.A lot of issues. I think at the start I, with a lot of other people, got their wires crossed. I thought that it would be like an education on what you could eat. Say, for instance, if you took a piece of Shepherd’s Pie, you could have salads and such and such instead of that, but it wasn’t what I thought. I did try it for 6 to 8 weeks, maybe not even that, and I was eating far more, far, far more. […] Plus, to me, it wasn’t healthy food, it wasn’t portion controlled.’*Female
Personal behaviour	Lack of control about personal diet	*‘Well, to be honest, at the start I was pretty upbeat about it but whenever it did start I felt it wasn’t what I had signed up for. I had signed up for more portion control and a lot more nutrition available, but me personally; I think it wasn’t what was discussed at the start. […] I was used to bringing in what I had in my lunch box, eating it and that was basically it. But it’s all probably about will power, you know, and you have a line of stuff, a wee bit of this, a wee bit of that and before you know it I was probably eating too much.’* Male
	Open to trying new food and would like to see a greater variety (this was reported by employees who didn’t take lunch and people who took lunch occasionally)	*‘Probably the soup, I love soup but I think a variety would be good. I know at the start there was butternut squash or carrot and lentil, and they’re all things that I wouldn’t have ate but with tasting them here I now like them ...I don’t like eating carrots by themselves but when they’re pureed and in the soup they’re tasty. […] I would like to see a bit more variety, I just know that variety is definitely the big thing. If you’re going to pay for something you’ll want to be enjoying it. You don’t want to be, “oh here we go; vegetable soup - again!” that sort of way.’* Female
	Repetitive food choices and reluctant to try new foods (found in people who took lunches)	
Menu	Menu was not displayed (not knowing what will be served)	*‘I probably would bring my lunch on the day, it all depends what was on [the menu]. Sometimes the menu is up and sometimes it isn’t.’* Female

Stakeholders overall felt that the food court has been a success and that the majority of employees benefited from it.

*‘I think overall it’s been very successful, in that employees that I talked to have all signed up to it, which are the vast majority. All say that they find it very beneficial, particularly from a health perspective but also from the fact that they have the choice and they have all agreed to take up the choice. So it’s been very positive all round.’ Stakeholder 1*

One issue that was brought up was that it was a challenge to please everyone.

*‘I think the numbers ties you slightly here as well because you’re down to 15 and if you’re going to split the choice at lunchtime it means that you have an awful lot of waste. It’s very difficult to get people pinned down. They’re not great at saying that they don't like something until suddenly you bring something different in and then they say "thank goodness, there’s no … ” whatever it is, you know?[…] it sort of does get more difficult to get the right choices on and things. They’re very conservative eaters here.’ Stakeholder 3*

The limited amount of resources put aside for the food court was also reported as a challenge:

*‘I suppose sometimes I feel that my hands are tied time wise and money wise, that there’s only a certain amount that I can dedicate it to it.’ Stakeholder 4*

Based on the feedback by staff and stakeholders, the research team made improvement suggestions to the management regarding improvement of the food court, its continuation and extension to the CS (Table 5).

**Table 5:** Suggestions made to management to improve the food court

• Allocate more preparation time and/ or budget to prepare the meals to ensure appropriate quality of food	
• Increase variety of foods, e.g. let staff choose between two or more options if the initiative is extended to the larger site	
• Reduce food waste, e.g. only provide food for people who indicate they would like to eat that day	
• Improve portion control, e.g. limit choice of foods on offer in one day (only the hot meal or sandwich + salads and fillings) and portion out hot food	
• Improve hygiene applied by all members of staff, e.g. brief staff on food handling and appropriate hygiene practices	
• Make weekly menu available to staff in advance	
• Communicate a clear rationale for the food court to employees eating lunch (recommend to focus on wholesomeness and healthiness of meals; minimal preparation time at home; opportunity for staff to interact)	

## Discussion

This was one of the first interventions to assess the introduction of the provision of free lunches to a worksite that represented a small workplace environment. The results showed small, significant results in SFA intake in the IS compared to the CS. No changes in F or V intake, other dietary measures, health or work-related measures were found. This study highlights the challenges that need to be considered when implementing an on-site catering facility free of charge for staff.

Research studies on dietary behaviour change interventions more frequently implement environmental changes to workplace canteens and it has been suggested that such changes can lead to positive effects in diet and health- related measures. An example where a workplace canteen intervention has significantly improved people’s diet habits was recently published by Lassen et al. [19]. They demonstrated that improving the nutritional quality of canteen meals improved F intake and reduced fat and energy consumption of health professionals. However, it is not a surprise that very small changes in diet and health-related measures were seen as part of the free lunch intervention, as there was a limited focus on the nutritional content of the lunches that were provided. This may have been a result of the lack of guidance from managers on the aim of the food court, who, when interviewed, had mixed views on the nutritional standards of the lunches that should have been provided.

Most of the dietary intervention studies published emphasised the healthy eating aspect to ensure the nutritional quality of the food provided [12, 20, 21]. Workplace diet interventions designed in line with dietary guidelines have been linked to positive changes in F and V consumption as well as improvements in other nutrients [3, 21, 22]. In contrast, the free lunch provision was only overseen by the managers and catering staff who were not trained nutrition experts. This highlights the importance of designing dietary interventions in line with dietary guidelines and working with trained experts to do so in order to maximise the chances of seeing improvements in dietary outcomes.

One limitation to the findings in diet outcomes is that all information was self-reported. Self-reported diet measures may be biased by under-reporting in the IS which may be indicated by the reduction in kcal in the IS, both, ON (− 114.7 kcal, SD: 581.4) and OFF duty (− 152.0, SD: 718.0) compared to the CS (− 94.5, SD: 561.8 and − 0.7 SD: 792.6 respectively). The 24-h diet recall does not reflect habitual diet intake and relied on the assumption that participants have similar eating habits most days. Although the 24-h diet recall may have limitations, it allows an insight into the differences in eating habits between weekend days and weekdays and adds important information to the literature, as this is a relatively unexplored topic.

Another limitation was the lack of clear guidance on the nutritional content of the lunches from management. Although the catering staff made an effort to serve nutritious lunches, due to limited time and resources and different eating preferences of staff, this was not always possible and may have contributed to the lack of long-term uptake and the small number of employees who had lunches from the food court at follow-up. Furthermore, the drop-out rate of the study was relatively high and further reduced the already initially small participant numbers. However, as the free lunch initiative was a pilot project, the information collected was valuable to inform a potentially larger project. The intervention was co-developed with the management and workforce, however, a theoretical basis was not considered. Workplace diet interventions may be more effective when based on theory [3], which should be considered for future interventions.

The fact that this was a controlled study means that any statistically significant changes seen were likely a result of the intervention. Although this study took place in a company employing over 300 staff, the intervention took place at a small worksite with only 20 staff that was separate to the rest of the company. Devine et al. suggested that especially small rural workplaces are overlooked in health promotion workplace interventions [23]. Therefore, this is one of the few studies that highlight the challenges of implementing diet interventions in smaller companies that currently offer no food provision on site. Even though not many companies will be able to afford to offer free lunches to their staff, lessons can be learned from this study with regards to challenges and facilitators to consider when implementing environmental interventions in small worksites. The qualitative feedback gives helpful insights into why this intervention did not have the desired effects in improving overall eating habits of staff and the barriers to successfully implementing the food court.

The findings suggest that future workplace interventions that aim to improve diet- and health-related measures need to strongly emphasise the importance of a well-balanced diet and nutritious meals to staff. Furthermore, decreasing the availability of unhealthy food and limiting portions sizes served may be important to achieve the desired improvements. As a free lunch intervention may not be realistic for most workplaces, employers should work with catering staff and employees to find a solution that is low-cost to implement and acceptable to staff to increase the likelihood of long-term sustainability. It would be of interest to compare the diet and health measures of workers who had the lunches compared to workers who did not have the lunches from the IS in a larger study. Comparing the meal composition of meals prepared at work compared to those prepared at home would also be of interest.

## Conclusion

The results from this pilot study suggest that the provision of the free lunches overall had little effect on employees’ F and V intake, overall dietary habits, health measures and job satisfaction. There was a lack of clear rationale and aim of the lunches from the management at the start of the introduction of the food court, which may be an explanation for the small change in outcome measures. Employees had mixed feelings on the service provided by the company. Involving employees in designing a healthy eating intervention at work and combining environmental components with education may improve staff buy-in and potentially lead to a more significant result in diet and health-related measures.

## Supplementary information

**Additional file 1.** Employee Topic Guide. This file contains the baseline and follow-up employee interview topic guide that was used to assess their motivation and views on healthy eating at work and the acceptability of the lunches as well as feedback on the food court at follow-up.

## Data Availability

The datasets used and/or analysed during the current study are available from the corresponding author on reasonable request.

## Abbreviations

BMI: Body mass index
CS: Control site
DBP: Diastolic blood pressure
F: Fruit
H: Hour
IS: Intervention site
NI: Northern Ireland
NMES: Non-milk extrinsic sugar
OFF duty: During the weekend
ON duty: During weekdays
SBP: Systolic blood pressure
SD: Standard deviation
SFA: Saturated fatty acid
UK: United Kingdom
V: Vegetables
WC: Waist circumference
