# Supplementary material for: Mixed methods evaluation of an employer-led, free lunch initiative in Northern Ireland
Source: BMC Nutr. 2019 Dec 18;5:60. doi: 10.1186/s40795-019-0321-8 (PMC7050844; doi:10.1186/s40795-019-0321-8)
Supplement: Supplementary file 1 — Additional file 1. Employee Topic Guide. This file contains the baseline and follow-up employee interview topic guide that was used to assess their motivation and views on healthy eating at work and the acceptability of the lunches as well as feedback on the food court at follow-up. [file 40795_2019_321_MOESM1_ESM.docx]

Topic guide: Focus Group Discussions Employees

The facilitator will introduce him/herself and reiterate the purpose of the interview i.e. to learn more about attitudes to healthy eating and eating at the workplace.

The facilitator will reiterate that the interview will be tape-recorded and transcribed for research purposes but transcripts will be anonymised i.e. will not contain participant names. Also the audio tapes will be destroyed when the typed transcripts have been prepared. In addition, it will be made clear to participants that they can inform the facilitator if there are any particular statements they do not wish to be transcribed at the end of the session.

The facilitator will ask the participant if they have any questions before starting the discussion.

- - *Can you describe what healthy eating means to you? (Prompt: Foods)*
  - *Are you aware of how diet can affect your health? (Prompt: Certain foods/ disease)*
  - *Are you concerned about what you eat and drink?*
  - *What influences what you eat and drink at home? (Prompts: Health concerns, Influences of other people, Availability, Cost, Cooking skills)*
  - *What influences what you eat and drink at work? (Prompts: Health concerns, Influences of other people, Availability, Cost, Time for breaks)*
  - *Do you feel you eat healthily eat work?*
  - *What would encourage you to eat more healthily at work? (Prompts: Availability, Cost)*
  - *Do you feel there are things that stop people from eating healthily at work and if so what are these? (Prompts: Canteen, vending machines, Local food shops/ Take-aways, Time to prepare lunch at home)*
  - *Do you feel it is important for your employer to provide healthy food and drink choices at work and to promote healthy eating? Why?*
  - *Do you think your employer currently helps to provide healthy food and drink choices at work and promote healthy eating?*

*(Probe: How do you feel about that the company does not have a canteen at the moment?)*

- - *If your employer wanted to make it easier for people to eat healthily at work, do you have any suggestions how that could be encouraged?*

*(Prompt: healthy lunch availability, vending machines, break time)*

*(Probe: How likely do you think people would make use of it?)*

- - *Is there anything else you would like to tell me or say about food and drink provision at work?*
- ***Follow-up:***
  - *How do you feel about the changes in the canteen? (Prompts: Availability of food, quality of food, social aspect of eating together, environment, time to eat)*
  - *Do you get lunch regularly in the canteen now – why/ why not? (Prompts: food choice and quality, convenience,* good variety, healthy choices, appealing, adequate portion sizes*)*
  - *Do you think your eating habits have changed since the food court has been introduced? (lunches, snacks)*
  - *Is there anything your employer could do to improve the canteen? (Prompt: Lunches, environment)*
  - *How well do you feel do people look after their own dishes? (everyone equally tidy, happy to look after themselves)*
